# Longitudinal Associations of Sedentary Behavior and Physical Activity With Diurnal Rest-Activity Rhythms in Survivors of Colorectal Cancer Up to 5 Years Post-Treatment

**DOI:** 10.1177/07487304251363674

**Published:** 2025-10-01

**Authors:** Koen G. Frenken, Marvin Y. Chong, Stéphanie O. Breukink, Maryska Janssen-Heijnen, Eric T.P. Keulen, Joop Konsten, Wouter Bijnens, Laurien M. Buffart, Kenneth Meijer, Frank A.J.L. Scheer, Karen Steindorf, Judith de Vos-Geelen, Matty P. Weijenberg, Martijn J.L. Bours, Eline H. van Roekel

**Affiliations:** * Department of Epidemiology, GROW Research Institute for Oncology and Reproduction, Maastricht University, Maastricht, The Netherlands; † Department of Epidemiology, CARIM Cardiovascular Research Institute Maastricht, Maastricht University Medical Center, Maastricht, The Netherlands; ‡ Department of Epidemiology, CAPHRI Care and Public Health Research Institute, Maastricht University, Maastricht, The Netherlands; § Department of Surgery, GROW Research Institute for Oncology and Reproduction, NUTRIM Research Institute of Nutrition and Translational Research in Metabolism, Maastricht University Medical Centre+, Maastricht, The Netherlands; || Department of Clinical Epidemiology, VieCuri Medical Centre, Venlo, The Netherlands; ¶ Department of Internal Medicine and Gastroenterology, Zuyderland Medical Centre Sittard-Geleen, Geleen, The Netherlands; # Department of Surgery, VieCuri Medical Centre, Venlo, The Netherlands; ** Research Engineering (IDEE), Maastricht University, Maastricht, The Netherlands; †† Department of Medical BioSciences, Radboud University Medical Center, Nijmegen, The Netherlands; ‡‡ Department of Nutrition and Movement Sciences, NUTRIM Research Institute of Nutrition and Translational Research in Metabolism, Maastricht University, Maastricht, The Netherlands; §§ Division of Sleep Medicine, Harvard Medical School, Boston, Massachusetts, USA; |||| Medical Chronobiology Program, Division of Sleep and Circadian Disorders, Departments of Medicine and Neurology, Brigham and Women’s Hospital, Boston, Massachusetts, USA; ¶¶ Division of Physical Activity, Prevention and Cancer, German Cancer Research Center (DKFZ), Heidelberg, Germany; ## Department of Internal Medicine, Division of Medical Oncology, GROW Research Institute for Oncology and Reproduction, Maastricht University Medical Centre, Maastricht, The Netherlands

**Keywords:** colorectal cancer survivorship, physical activity, sedentary behavior, diurnal rest-activity rhythms, circadian quotient, dichotomy index, 24-h autocorrelation, acrophase

## Abstract

Disrupted diurnal rest-activity rhythms (RAR), that is, daily 24-h patterns of rest and activity, have been associated with fatigue and decreased quality of life among survivors of colorectal cancer (CRC). To identify potential targets for interventions to improve RAR, we investigated longitudinal associations of time spent in sedentary behavior and physical activity with RAR parameters after CRC treatment. In a prospective cohort study, repeated measurements were performed among 268 survivors of stage I–III CRC at 6 weeks, 6 months, and 1, 2, and 5 years after treatment. Thigh-worn accelerometers were used to determine hours/day spent in sedentary behavior, standing, and total physical activity during waking time, as well as RAR parameters including mesor, amplitude, circadian quotient (CQ), dichotomy index (I < O) and 24 h-autocorrelation (R24). Self-reported light-intensity physical activity (LPA) and moderate-to-vigorous physical activity (MVPA) were determined via the validated SQUASH questionnaire. Longitudinal associations were analyzed using confounder-adjusted linear mixed models. More sedentary time was statistically significantly associated with a lower mesor, amplitude, I < O and R24 over the 5-year post-treatment period. More standing time was associated with a higher mesor, amplitude, CQ, and I < O but not with R24. Higher levels of objectively assessed total physical activity as well as self-reported MVPA were associated with higher values for all RAR parameters. LPA was not associated with any of the RAR parameters. In the years after CRC treatment, less sedentary behavior and more standing and physical activity were generally associated with higher RAR parameters indicating a more robust rhythm. Future studies should provide more insight into causality of these associations as RAR may be a potential new target for interventions to reduce fatigue after CRC.

Trial registration: EnCoRe study NL6904 (https://www.Onderzoekmetmensen.nl/).

Colorectal cancer (CRC), being the third most diagnosed cancer type, is increasing in incidence due to an aging population, unhealthier lifestyles and improved screening (Mármol et al., 2017, Oruç and Kaplan, 2019). Fortunately, the survival rate is rising due to improved treatment options and improved secondary preventive strategies (Arnold et al., 2017). Therefore, more patients are living with long-term side-effects after cancer including fatigue, diminished quality of life, depression, and sleep problems (i.e. insomnia) (Hrushesky et al., 2009, Rawla et al., 2019, Amidi and Wu, 2022). Notably, chronic fatigue has been observed in more than 30% of CRC survivors (Thong et al., 2013, Vardy et al., 2016, Xian et al., 2021).

Evidence is emerging that diurnal rest-activity rhythm (RAR) disruptions may play an important role in cancer- and treatment-related symptoms, such as fatigue (Rich, 2007, Milanti et al., 2021, Amidi and Wu, 2022). RAR represents the daily pattern of rest and activity over a 24-h period. This can be measured objectively using accelerometers that assess parameters such as the mesor (mean activity levels), amplitude (peak and mean activity difference), acrophase (clock time of peak activity), circadian quotient (CQ; amplitude adjusted for the mesor), dichotomy index (day-night activity difference), and the 24-h autocorrelation coefficient (rhythmic consistency across days) (Fernandes et al., 2006, Milanti et al., 2021). These parameters are measures of the robustness of the RAR, except for acrophase, where the lower tertiles indicate a more disrupted rhythm while the middle tertile values represent a more robust rhythm. For the acrophase, a later timing has been correlated to poorer global quality of life (Bernatchez et al., 2018).

Research investigating shift work also indicates that a more disrupted rhythm is associated with higher cancer incidence, faster progression, and shorter survival in breast and endometrial cancer (Schernhammer et al., 2001, Fernandes et al., 2006, Viswanathan et al., 2007, Hrushesky et al., 2009, Milanti et al., 2021). In addition, a more disrupted RAR is associated with more fatigue, anxiety and depression, impaired sleep, and poorer quality of life in patients with lung, colon, ovarian and breast cancer, and survivors of CRC (Davis et al., 2001, Hrushesky et al., 2009). Disruption of RAR was found to be an independent prognostic factor of both progression-free and overall survival in patients with metastatic CRC (Mormont et al., 2000, Innominato et al., 2009, Lévi et al., 2014). Supporting these findings, our research group previously found survivors of stage I–III CRC with a more pronounced RAR experienced lower fatigue and insomnia, and a better quality of life during 5 years after treatment (Chong et al., 2024).

Potential actionable parameters to enhance RAR robustness are changing physical behaviors such as sedentary behavior, standing, and physical activity (Vitaterna et al., 2001, Lévi et al., 2020, Shen et al., 2023). The link between sedentary behavior and physical activity in relation to fatigue and quality of life within cancer survivors is well-established (Lynch et al., 2013, Blair et al., 2014, Sylvester et al., 2017, Campbell et al., 2019, Dun et al., 2020, Mazzoni et al., 2023). However, to our knowledge, it has not been investigated to date whether and how sedentary behavior and physical activity are related to RAR in any cancer survivorship population. Due to the inherent relation between physical activity and some RAR variables such as the mesor, it is likely that as physical activity is increased, RAR parameters will also be improved. At the same time, for other parameters such as R24 and acrophase this relationship is less clear. Therefore, the aim of this study is to investigate how sedentary behavior, standing, and physical activity are longitudinally associated to RAR parameters in CRC survivors, from 6 weeks until 5 years after treatment. We hypothesized that higher sedentary behavior and lower standing and physical activity are associated with lower RAR parameters indicating a less robust RAR.

## Methods

### Study Design and Population

We used data from the Energy for life after ColoRectal cancer (EnCoRe) study, a prospective cohort study of survivors of stage I–III CRC in the south of the Netherlands (Netherlands Trial Register number: NL6904). Since 2012, participants have been recruited from three hospitals (Maastricht University Medical Center+, VieCuri Medical Center, and Zuyderland Medical Center). Participants were excluded if they were diagnosed with stage IV CRC, were below the age of 18, were unable to understand or speak the Dutch language, resided outside of the Netherlands or had comorbidities that could hinder participation (e.g. a cognitive disorder, or problems with hearing or visibility). In addition, we excluded data from individuals who did not have available data from the MOX accelerometer on sedentary behavior, physical activity, and diurnal RARs. Data were obtained by trained personnel during repeated measurements at the participants’ homes or via postal mail. These repeated measurements were performed at 6 weeks, 6 months, 12 months, 24 months, and 60 months after the end of the treatment. For the current analyses, data up to July 2018 were used for the 6-week to 24-month time points. For the measurements at 60 months post-treatment, data collected until October 2021 were included. A total of n = 268 participants were included at 6 weeks post-treatment, n = 254 at 6 months, n = 214 at 12 months, n = 138 at 24 months and n = 77 at 60 months post-treatment.

**Figure 1.**
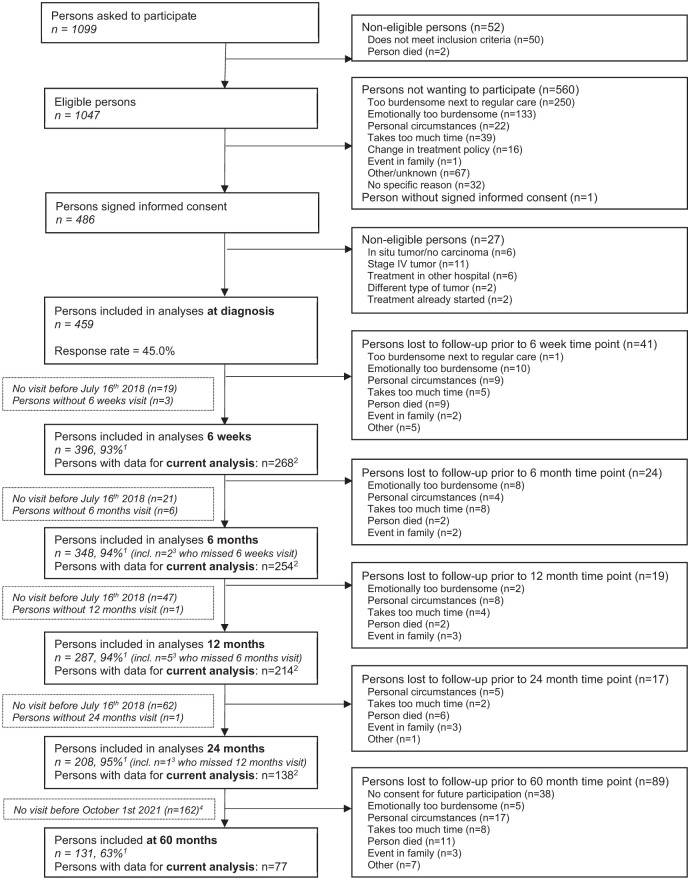
Flow diagram of the inclusion of participants within the Energy for Life after ColoRectal cancer (EnCoRe) study from 2012 onwards and the number of post-treatment measurements up to July 2018 for the 6-week to 24-month time points and up to October 2021 for the 60-month time points included in the analyses of the current paper. ^1^Response rate = (persons with measurements)/(persons with measurements + persons lost to follow-up - persons died). The declining number of participants at subsequent time points is largely because not all participants included at diagnosis had reached all follow-up time points by July 2018 (6 weeks until 24 months post-treatment) or October 2021 (60 months post-treatment). ^2^Since the analyses in this article were focused on sedentary behavior, physical activity, and diurnal rest-activity rhythms, only post-treatment measurements with available data on these parameters were included. ^3^Of the three persons without 6-week follow-up visits, one person did not have a 6-month follow-up visit before July 16, 2018. Of the six persons without 6-month follow-up visits, one person did not have a 12-month follow-up visit before July 16, 2018. ^4^All data collected from participants between April 2012 and July 2018 were used for the measurements from 6 weeks until 24 months post-treatment. These participants were followed over time and data collected up to October 2021 was used for the 60 months post-treatment follow-up measurement.

This study was approved by the Medical Ethics Committee of the University Hospital Maastricht and Maastricht University (METC 11-3-075). All participants provided written informed consent. A flow diagram describing the inclusion of the patients within the EnCoRe study and in the current analyses can be found in Figure 1.

### Objectively Assessed Sedentary Behavior and Physical Activity Parameters

Sedentary behavior, standing, and physical activity were objectively assessed and categorized using the validated tri-axial MOX activity monitor (MMOXX1, upgraded version of the CAM; Maastricht Instruments B.V., Maastricht, The Netherlands) (Annegarn et al., 2011, Berendsen et al., 2014). The activity monitor was worn on the right anterior upper thigh, approximately 10 cm above the knee for seven consecutive days (24 hours/day) during every post-treatment time point. The MOX activity monitor measures raw acceleration data in three orthogonal sensor axes at a sampling rate of 25 Hz. Data on hours/day of total sedentary behavior and hours/day spent in prolonged sedentary bouts defined as uninterrupted bouts of sedentary behavior (sitting or lying during waking hours at a low intensity of ≤1.5 METs (metabolic equivalence of task)) of at least 30 minutes (Chastin and Granat, 2010, van Roekel et al., 2016b, Tremblay et al., 2017, Bellettiere et al., 2021), were used in the current analyses. Standing time was defined as the total daily time in any waking activity in a standing posture characterized by an energy expenditure of ≤1.5 METs (Tudor-Locke et al., 2014, Tremblay et al., 2017). Total physical activity was defined as any movement or posture during waking hours that exceeded 1.5 METs (Mendes et al., 2018). Waking time and bedtimes were self-reported by participants in a structured 7-day dietary record and in case of discrepancies checked with the MOX accelerometer. All objectively assessed variables were calculated for every valid day and subsequently averaged across all days available at each post-treatment time point. Measurements days were considered valid when there was 24-hour wear time (i.e. no non-wear). In addition, only measurements with at least four valid days including one weekend day were included in the current analysis. This meant a total of 7.4% of total measurements were excluded.

A custom-made MATLAB program (version R2022a; The MathWorks, Inc., Natick, MA) was used to process accelerometer data and assess hours/day of prolonged sedentary behavior, standing, and total physical activity (van Roekel et al., 2016a). The MOX accelerometer was shown to have moderate to high reproducibility and high validity to assess sedentary behavior (Berendsen et al., 2014). Although the device validly measures total physical activity, the monitor has limited reproducibility for distinguishing between different intensity levels of physical activity (Troiano et al., 2008, Berendsen et al., 2014).

### Self-Reported Physical Activity

The Short QUestionnaire to ASsess Health-enhancing physical activity (SQUASH) is a validated physical activity questionnaire to determine self-reported time spent in light-intensity physical activity (LPA) and moderate-to-vigorous physical activity (MVPA) (Wendel-Vos et al., 2003, Nicolaou et al., 2016). Participants reported time spent on commuting, household, work, and leisure time activities throughout the week. Based on Ainsworth’s Compendium of Physical Activities, all activities were assigned MET values (Ainsworth et al., 1993). Total weekly LPA and MVPA (hours/week) was determined by summing the time spent in activities with an energy expenditure of 1.5 to 3.0 METs and >3.0 METs, respectively (Mendes et al., 2018, Menezes-Júnior et al., 2023). Both LPA and MVPA were subsequently converted to average hours/day, for consistency with accelerometer data for which also average hours/day were calculated. The SQUASH was shown to be reliable (test–retest: Spearman’s ρ = 0.57-0.58) (Wendel-Vos et al., 2003, Wagenmakers et al., 2008). Relative validity, in comparison to an accelerometer, was found to be comparable (Spearman’s ρ = 0.40 for moderate-intensity activities) with other physical activity questionnaires (Wendel-Vos et al., 2003).

### Diurnal RARs

The MOX accelerometer was also used to determine parameters of RAR. The acceleration data that were collected were converted into activity counts in 1-minute epochs (intervals), using the signal magnitude area (Bijnens et al., 2019). The epochs were used for determining RAR parameters as described below.

**Figure 2.**
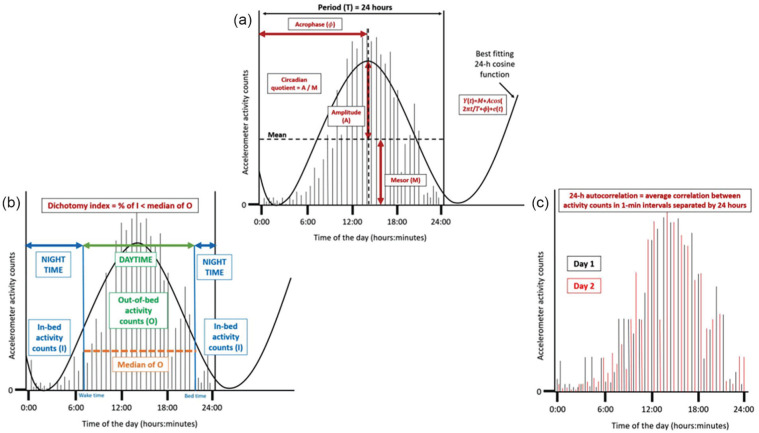
Visualization of the operationalized diurnal rest-activity rhythm parameters mesor, amplitude, acrophase (in hh:mm), circadian quotient, dichotomy index and the 24-h autocorrelation coefficient, based on fictitious accelerometer data (Chong et al., 2024). Panel A shows the acrophase, circadian quotient, amplitude and mesor. Panel B shows the dichotomy index and Panel C shows the 24-h autocorelation.

RAR parameters included mesor, amplitude, acrophase, CQ, dichotomy index (I < O), and 24-h autocorrelation (R24). These parameters were calculated as part of the custom-made MATLAB program that was used to process accelerometer data as described above (Maastricht Instruments B.V., Maastricht, The Netherlands). Figure 2 shows how the parameters were operationalized and how they can be interpreted (Chong et al., 2024). All parameters besides the R24 were calculated for every valid day, and subsequently averaged across all days available at each post-treatment time point. For the R24, one value for each post-treatment time point was obtained based on all measurement days. The cosinor method, which is widely used in RAR studies, was used to obtain the mesor, amplitude, and acrophase (Halberg and Katinas, 1973, Nelson et al., 1979).

#### Mesor

The midline estimating statistic of the rhythm (mesor) is the mean of the activity counts across the 24-h day. Higher values demonstrate more activity across the 24-h day (Lentz, 1990, Miaskowski et al., 2011).

#### Amplitude

The amplitude is the difference between the highest activity peak of the cosinor curve and the mesor in activity counts. Higher values for amplitude indicate a larger contrast between average activity levels and peak activity (Lentz, 1990, Miaskowski et al., 2011, Berger et al., 2012).

#### Acrophase

The acrophase describes the clock time of the cosine weighted peak and is expressed in decimal hours. Acrophase is often an appropriate estimate of the timing of a person’s 24-h rhythm (i.e. being more active earlier or later in the day) as it describes the clock timing of the peak activity (Lentz, 1990).

#### Circadian Quotient

The CQ is determined by dividing the amplitude by the mesor. The advantage of this measure is that, unlike the amplitude, it is a measure that corrects the amplitude for the average activity. Higher values, that is, higher amplitude relative to mesor, reflect a stronger RAR (Berger et al., 2012).

#### Dichotomy Index

The dichotomy index describes the proportion of in-bed (I) activity counts that are less than the median of out-of-bed (O) activity counts (Natale et al., 2015). The I < O was calculated for each 24-h period ranging from 00:00 to 23:59 h, on all valid wear days (Natale et al., 2015). Higher I < O values indicate a stronger RAR (Natale et al., 2015).

#### Twenty-Four-Hour (24 h) Autocorrelation

The 24-h autocorrelation coefficient quantifies the consistency of the RAR from 1 day to the next. This parameter describes the average correlation between activity count levels in 1-min epochs separated by 24 h. The 24-h autocorrelation was only calculated in the case of seven valid wear days. Due to non-wear on one or more days, 31% of accelerometer measurements were excluded for this parameter as compared to the other RAR parameters. Higher and positive values for the 24-h autocorrelation coefficient indicate a more robust RAR (Berger et al., 2012).

### Sociodemographic, Lifestyle, and Clinical Factors

Sociodemographic characteristics including age (years), sex (male/female), and education level (low, medium or high see Table 1 footnote c) were self-reported at diagnosis. Smoking status (current, former or never), employment status (job/no job), and the presence of a stoma were self-reported at diagnosis and at each post-treatment measurement. Body mass index (BMI; kg/m^2^) was calculated at baseline and at post-treatment measurements based on measured body height and weight. BMI was categorized using the World Health Organization (WHO) guidelines into underweight (BMI < 18.5 kg/m^2^), normal weight (18.5 ≤ BMI < 25 kg/m^2^), overweight (25 ≤ BMI < 30 kg/m^2^), or obese (BMI ≥ 30 kg/m^2^) (Organization, 2011). Dietary intake was measured via a 7-day food diary at each of the post-treatment time points and (among others) used to determine alcohol consumption (g/day). The number of comorbidities (0, 1, or ≥2) was assessed using the 13-item administered Comorbidity Questionnaire, at each post-treatment measurement (Sangha et al., 2003). Clinical data were collected from medical records and included cancer stage (I, II, or III), tumor site (colon or rectosigmoid and rectum) and treatment type (surgery, chemotherapy, and/or radiotherapy).

**Table 1.** Sociodemographic, lifestyle, and clinical characteristics in the study population of CRC survivors.

Characteristics	6 weeks post-treatment (n = 268)^a^	6 months post-treatment (n = 254)^a, b^	12 months post-treatment (n = 214)^a, b^	24 months post-treatment (n = 138)^a, b^	60 months post-treatment (n = 77)^a, b^
Age (years), mean ± SD	66.8 ± 8.9	67.3 ± 8.8	66.8 ± 8.8	68.7 ± 9.2	70.4 ± 9.2
Sex (male), *n* (%)	179 (66.8)	173 (68.0)	142 (66.4)	93 (67.4)	50 (64.9)
Education level, *n* (%)^c^					
Low	72 (26.9)	68 (26.8)	51 (23.8)	30 (21.7)	16 (20.8)
Medium	102 (38.1)	103 (40.6)	84 (39.3)	58 (42.0)	28 (36.4)
High	94 (35.1)	83 (32.7)	79 (36.9)	50 (36.2)	33 (42.9)
BMI (kg/m^2^), mean ± SD	27.5 ± 4.4	28.0 ± 4.5	28.5 ± 4.8	28.4 ± 4.6	27.2 ± 4.5
BMI categorical, *n* (%)					
Underweight (<18.5 kg/m^2^)	2 (0.8)	0 (0.0)	1 (0.5)	1 (0.7)	1 (1.3)
Healthy weight (18.5-24.9 kg/m^2^)	84 (31.3)	66 (26.0)	50 (23.4)	30 (21.7)	27 (35.1)
Overweight (25.0-29.9 kg/m^2^)	110 (41.0)	114 (44.9)	94 (43.9)	60 (43.5)	26 (33.8)
Obese (≥30 kg/m^2^)	72 (26.9)	74 (29.1)	69 (32.2)	47 (34.1)	23 (29.9)
Smoking status, *n* (%)					
Current	23 (8.6)	18 (7.1)	22 (10.3)	10 (7.3)	8 (10.4)
Former	161 (60.1)	160 (63.0)	130 (60.8)	85 (61.6)	47 (61.0)
Never	84 (31.3)	76 (29.9)	62 (29.0)	43 (31.2)	22 (28.6)
Alcohol intake (g/day), median (IQR)	6.1 (0.0 – 19.5)	5.9 (0.0 – 21.7)	8.7 (0.2 – 22.8)	7.8 (0.0 – 19.3)	7.2 (0.0 – 21.7)
Employment (yes), *n* (%)	82 (30.6)	68 (26.8)	59 (27.6)	29 (21.0)	15 (19.5)
Stoma (yes), *n* (%)	74 (27.6)	43 (16.9)	29 (13.6)	16 (11.6)	10 (13.0)
Treatment, *n* (%)					
Surgery (yes)	237 (88.4)	228 (89.8)	189 (88.3)	123 (89.1)	69 (90.0)
Chemotherapy (yes)	101 (37.7)	93 (36.6)	82 (38.3)	54 (39.1)	25 (32.5)
Radiotherapy (yes)	70 (26.1)	67 (26.4)	57 (26.6)	39 (28.2)	20 (26.0)
Tumor site, *n* (%)					
Colon	86 (32.1)	73 (28.7)	69 (32.2)	44 (31.9)	23 (29.9)
Sigmoid	81 (30.2)	87 (34.3)	65 (30.4)	38 (27.5)	24 (31.2)
Rectum	101 (37.7)	94 (37.0)	80 (37.4)	56 (40.6)	30 (39.0)
Cancer stage, *n* (%)					
Stage I	85 (31.7)	83 (32.7)	72 (33.6)	44 (31.9)	24 (31.2)
Stage II	68 (25.4)	61 (24.0)	50 (23.4)	36 (26.1)	25 (32.5)
Stage III	115 (42.9)	110 (43.3)	92 (43.0)	58 (42.0)	28 (36.4)
Comorbidities, *n* (%)					
0	53 (19.8)	61 (24.0)	57 (26.6)	34 (24.6)	25 (32.5)
1	71 (26.5)	68 (26.8)	46 (21.5)	36 (26.1)	22 (28.6)
≥2	144 (53.7)	125 (49.2)	111 (51.9)	68 (49.3)	30 (39.0)

Abbreviations: BMI, body mass index; SD, standard deviation; IQR, interquartile range.

a. Percentages may not add up to 100% due to rounding.

b. Response rates for the follow-up time points up to 24 months were all above 90% and for 60 months 63%. The decreasing absolute numbers are largely due to the fact that participants had not yet reached all post-treatment follow-up time points at the time of data acquisition in July 2018 for measurements at 6 weeks to 24 months post-treatment and in October 2021 for 60 months post-treatment.

c. Highest attained education, low: no education, primary education, or basic vocational education; medium: advanced vocational education or senior secondary vocational education; high: senior secondary general education, higher professional education, or academic higher education.

### Statistical Analysis

Descriptive analyses were performed to summarize sociodemographic, lifestyle and clinical characteristics, sedentary behavior, standing and physical activity parameters, and the RAR parameters at each post-treatment time point. Quantitative variables that were normally distributed were presented as mean (±SD), whereas non-normally distributed quantitative variables were presented as median with interquartile range (IQR). Categorical variables were described as frequencies with percentages across categories. For describing longitudinal changes over time in sedentary, standing, and physical activity behavior and RAR parameters, linear mixed-model regression analysis was applied with time modeled as a continuous and categorical variable (i.e. indicator variables per time point).

Pearson’s correlation coefficients were calculated at 6 weeks post-treatment to determine the correlation between sedentary behavior and physical activity variables and the RAR parameters. Linear mixed models were used to analyze longitudinal associations of sedentary, standing and physical activity behavior with RAR parameters from 6 weeks to 60 months post-treatment. This method accounts for the correlation of repeated measures within individuals by incorporating random intercepts and a random slope if it improved the model fit (Twisk, 2013). Linear mixed models are a capable of handling missing data that are missing at random (MAR) or missing completely at random (MCAR) (JWR, 2013). Detailed information about linear mixed models can be found elsewhere (Gilbert, 2008, Chiu et al., 2019, Wang et al., 2022). Total sedentary time and prolonged sedentary time were analyzed per 2 hours per day, while all other variables (standing time, total physical activity, LPA and MVPA) were analyzed per 1 hour per day, being comparable to the SD of these variables. All RAR parameters were standardized by dividing individual values by the mean standard deviation across all five post-treatment time points for each parameter to enable comparison of regression coefficients between different parameters. The mesor, amplitude, CQ, dichotomy index and 24 h autocorrelation were modeled continuously.

The relation between sedentary behavior and physical activity with acrophase was modeled using longitudinal multinomial logistic regression with the acrophase categorized into tertiles. This model was used since we expected a potential non-linear relationship between sedentary behavior and physical activity with this outcome. The resulting odds ratios (ORs; also referred to as relative risk ratios) represent the likelihood of being in a certain acrophase tertile (e.g. third tertile) relative to the reference category (e.g. first or second tertile), per unit alteration change in physical activity or sedentary behavior variables (e.g. 2 hours/day more sedentary behavior). As 98% of the acrophases (peak clock times) were contained within 6:00 AM to 6:00 PM, we used tertiles of acrophases rather than a 24-h approach. Since the linearity assumption was not met, we grouped the acrophases. Tertiles were chosen over quartiles or deciles to ensure sufficient sample sizes in each group maximizing statistical power. Dividing the patients into larger groups would have therefore resulted in groups that would have been too small. Acrophase was categorized using tertiles into an early group (tertile 1 hh:mm:ss = 01:23:35-13:51:20), mid-day group (tertile 2 = 13:51:28-14:42:40) and later group (tertile 3 = 14:42:55-20:10:02) as previously done in literature (Vitale et al., 2015, Yi Lee et al., 2021). Tertiles were only used for the variable acrophase.

Longitudinal associations were adjusted for confounders, defined a priori and identified through existing literature (Lemmens et al., 2005, Gong et al., 2012, Kim et al., 2015, Boakye et al., 2019, Chong et al., 2024, Liu et al., 2024). Fixed (time invariant) confounders included age at enrollment (years), sex, neoadjuvant therapy (chemo and/or radiotherapy: yes, no), adjuvant chemotherapy (yes, no), and education level (low, medium, high). Time-variant confounders, which were measured at all post-treatment time points, included number of comorbidities (0, 1, ≥2), BMI (kg/m^2^), stoma (yes, no), smoking (current, former, never), employment status (employed, unemployed/retired), alcohol intake (g/day), and time since end of treatment (days). A likelihood-ratio test was used to evaluate whether a random slope was necessary to improve the model fit. Next to analyzing overall longitudinal associations, inter- and intra-individual associations were disaggregated by adding centered person mean values to the model to estimate inter-individual associations (i.e. average differences between participants over time) and individual deviations from the person-mean value to estimate intra-individual associations (i.e. within-participant changes over time) (Twisk and de Vente, 2019). Due to the nature of the multinomial logistic regression model used, for the acrophase, no inter- and intra-individual associations were calculated.

Effect modification by sex, BMI (continuous), and time since end of treatment (categorical per time point), in relation to RAR parameters, were examined by adding interaction terms into the linear mixed models. Significance was set at *p* < 0.05 for interaction terms.

To obtain more insight into the possible direction of longitudinal associations, we performed a sensitivity analysis using time lag models, in which sedentary behavior and physical activity variables at earlier time points were coupled with RAR parameters at subsequent time points to mimic a more natural direction of associations. Three additional sensitivity analyses including additional adjustment for potential additional confounders were performed, including self-reported napping during the day (yes/no), additional adjustment of models with (prolonged) sedentary behavior, LPA and standing time as independent variables for MVPA, and adjustment of models with MVPA as an independent variable for total sedentary behavior. All statistical analyses were performed using Stata 16.0 (StataCorp LLC) with statistical significance set at *p* < 0.05 (two-sided). Sensitivity analyses were displayed as comparison plots made in R-Studio 2024.09.0 + 375.

## Results

About two-thirds of the participants were men (67%), and participants had an average age of 67 years (SD = 9) at 6 weeks post-treatment (baseline for the longitudinal analysis - Table 1). At baseline participants consumed 6.1 (0.0-19.5) g/day of alcohol, there were a total of 23 (8.6%) smokers, and BMI was 27.5 ± 4.4 kg/m^2^ and remained stable over time. 32% of participants were survivors of a colon tumor, 30% of a sigmoid tumor and 38% of a rectum tumor. Around 88% of survivors underwent surgery and 38% underwent chemotherapy. A large portion of participants were diagnosed with stage III CRC (43%), and more than half of all participants had ≥2 comorbidities (54%) at 6 weeks post-treatment.

### Descriptives of Sedentary Behavior and Physical Activity Variables and Diurnal RAR Parameters Up to 60 Months Post-Treatment

**Figure 3.**
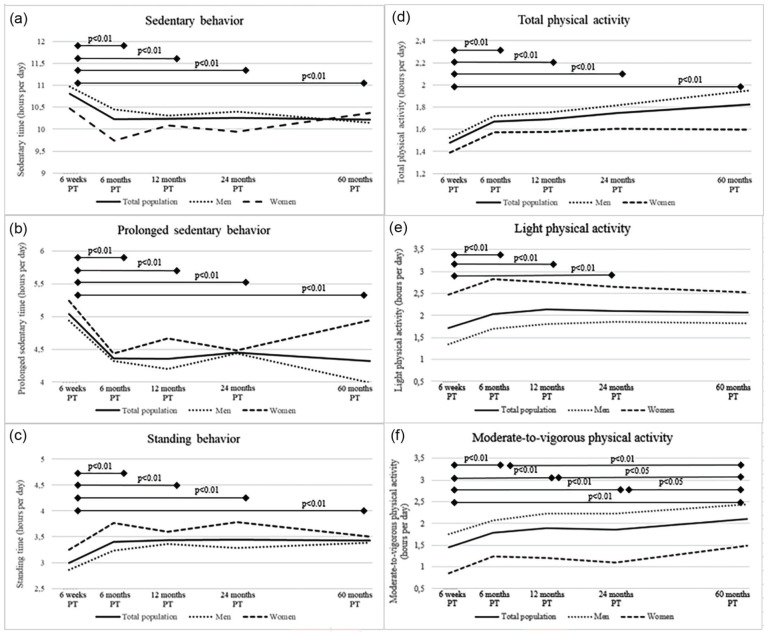
Course of mean sedentary behavior and physical activity variables (a, sedentary time; b, sedentary bouts; c, standing time; d, total physical activity; e, light physical activity; and f, moderate-to-vigorous physical activity) from 6 weeks up to 60 months post-treatment in colorectal cancer survivors included in the EnCoRe study. Variables depicted in panels a-d are measured via the MOX accelerometer while variables in panels e and f are measured by the SQUASH questionnaire. P values were obtained for the difference between follow-up time points for the total population via linear mixed models. They were conducted with sedentary behavior and physical activity variables as outcome variable and time modeled as independent categorical variables represented by dummy variables. Measures of variability can be found in Table 2.

At 6 weeks post-treatment, total (mean: 10.8 ± SD: 1.7 h/day) and prolonged (5.0 ± 2.4 h/day) sedentary behavior were at its highest (Table 2). Standing (3.0 ± 1.0 h/day), total physical activity (1.5 ± 0.6 h/day), LPA (median: 1.1; IQR: 0.3-2.5) h/day) and MVPA (1.0; 0.5-2.1 h/day) were lowest at 6 weeks post-treatment. Sedentary, standing and physical activity behavior, except for LPA, were statistically different at 6 weeks post-treatment compared to the other post-treatment time points (Figure 3). P-values were noted to indicate statistical significance of the change between time points. Descriptives further indicated that men generally had more MVPA than women while women had a higher LPA.

**Table 2.** Descriptive analyses of RAR parameters and sedentary behavior, standing and physical activity variable in the study population of CRC survivors.

Characteristics	6 weeks post-treatment (N = 268)	6 months post-treatment (N = 254)^a^	12 months post-treatment (N = 214)^a^	24 months post-treatment (N = 138)^a^	60 months post-treatment (N = 77)^a^
Sedentary behavior and physical activity variables (mean ± SD)/(median: 25th percentage-75th percentage) (hours/day)					
Sedentary behavior	10.8 ± 1.7	10.2 ± 1.5	10.2 ± 1.5	10.3 ± 1.4	10.2 ± 1.5
Prolonged sedentary behavior	5.0 ± 2.4	4.4 ± 2.0	4.4 ± 1.9	4.4 ± 1.9	4.3 ± 2.0
Standing behavior	3.0 ± 1.0	3.4 ± 1.1	3.5 ± 1.1	3.4 ± 1.0	3.4 ± 1.0
Total physical activity	1.5 ± 0.6	1.7 ± 0.6	1.7 ± 0.6	1.7 ± 0.7	1.8 ± 0.8
LPA	1.1 (0.3 - 2.5)	1.5 (0.5 - 3.0)	1.5 (0.4 - 3.0)	1.5 (0.5 - 3.0)	1.5 (0.5 - 2.5)
MVPA	1.0 (0.5 - 2.1)	1.4 (0.8 - 2.4)	1.5 (0.8 - 2.6)	1.4 (0.9 - 2.6)	1.6 (1.0 - 2.7)
Diurnal rest activity parameters (mean ± SD)/(median: 25th percentage-75th percentage)					
Mesor	3.7 ± 0.2	3.8 ± 0.2	3.8 ± 0.2	3.8 ± 0.2	3.8 ± 0.2
Amplitude	0.6 ± 0.2	0.6 ± 0.2	0.6 ± 0.2	0.6 ± 0.2	0.6 ± 0.2
Acrophase (clock time in hh: mm)	14:14 (13:30 - 14:58)	14:18 (13:43 - 14:57)	14:22 (13:41 - 14:57)	14:12 (13:27 - 14:56)	14:22 (13:55 - 14:53)
Circadian quotient	0.2 ± 0.0	0.2 ± 0.0	0.2 ± 0.0	0.2 ± 0.0	0.2 ± 0.0
Dichotomy index	0.8 ± 0.2	0.9 ± 0.1	0.9 ± 0.1	0.9 ± 0.1	0.9 ± 0.1
24 h—auto correlation^b^	0.2 ± 0.1	0.2 ± 0.1	0.2 ± 0.1	0.2 ± 0.1	0.2 ± 0.1

Abbreviations: LPA, light-intensity-physical-activity; MVPA, moderate-to-vigorous-intensity-physical-activity; SD, standard deviation; IQR, interquartile range.

a. Response rates for the follow-up time points up to 24 months were all above 90% and for 60 months 63%. The decreasing absolute numbers are largely due to the fact that participants had not yet reached all post-treatment follow-up time points at the time of data acquisition in July 2018 for measurements at 6 weeks to 24 months post-treatment and in October 2021 for 60 months post-treatment

b. To have a representative number based on the full 7 day week, the 24-h autocorrelation coefficient was only calculated in case of seven valid wear days. Therefore, there were fewer accelerometer measurements available for this parameter at 6 weeks post-treatment (n = 189), at 6 months post-treatment (n = 177), at 12 months post-treatment (n = 149), at 24 months post-treatment (n = 110), and at 60 months post-treatment (n = 41).

**Figure 4.**
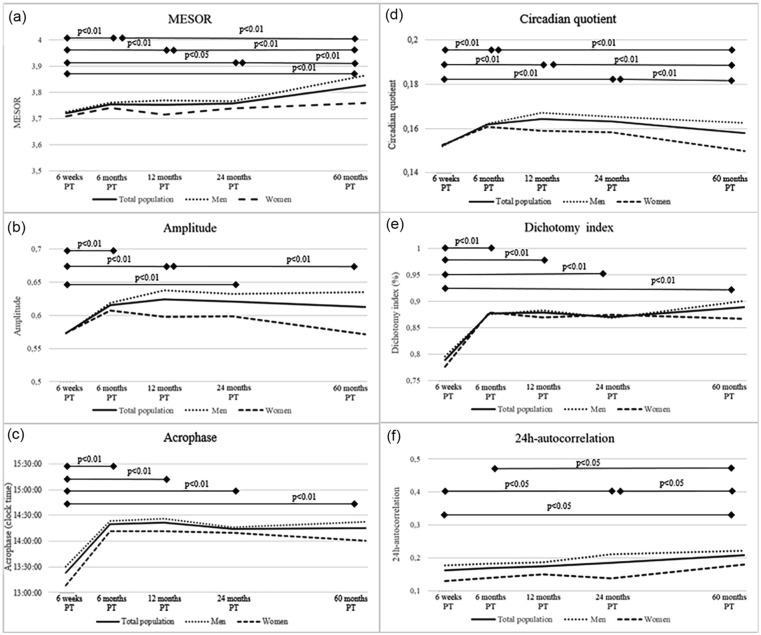
Course of mean diurnal rest-activity parameters (a, Mesor; b, amplitude; c, acrophase; d, circadian quotient; e, dichotomy index; and f, 24-h autocorrelation) from 6 weeks up to 60 months post-treatment in colorectal cancer survivors included in the EnCoRe study. *P* values were obtained for differences between follow-up time points for the total population via linear mixed models. They were conducted with diurnal-rest activity parameters as outcome variable and time modeled as independent categorical variables represented by dummy variables. Higher values on the various parameters indicate a more pronounced activity rhythm besides the acrophase where there is an ideal range. Measures of variability can be found in Table 2.

At 6 weeks post-treatment, the mesor was 3.7 ± 0.2 counts/day, the amplitude 0.6 ± 0.2 counts/day, the acrophase 14:14 h (IQR: 13:30 h-14:58 h), the CQ 0.2 ± 0.0 per day, the dichotomy index 0.8 ± 0.2 per day, and finally, the R24 0.2 ± 0.1 per day (Figure 4). The mesor, acrophase and I < O were significantly different from 6 weeks post-treatment to all other post-treatment time points, while other parameters remained stable. *P* values were noted to indicate statistical significance of the change between time points. No major sex differences were observed.

Weak to strong correlations were found between RAR parameters and total physical activity (range in Pearson’s r: 0.04, 0.75) (Supplementary Figure 1). Sedentary and prolonged sedentary behavior had weak to strong negative correlations with all RAR parameters (r: −0.76, −0.18). Standing behavior and MVPA showed to have weak to moderate correlations (r: 0.01, 0.58) while LPA had weak correlations with RAR parameters (−0.12, 0.16). The RAR parameters with one another showed that the amplitude was highly correlated to the CQ (r: 0.99) as well as the amplitude with the mesor (r: 0.75). In addition, I < O was seen to be highly correlated with the acrophase (r: 0.79). The rest of the RAR parameters were weak to strongly correlated: (r: −0.04, 0.69).

## Longitudinal Associations of Sedentary Behavior and Physical Activity with Diurnal Rars

### Sedentary Behavior and Prolonged Sedentary Behavior

Higher values for objectively assessed sedentary behavior and prolonged sedentary behavior were statistically significantly associated with lower values for all RAR parameters besides the R24 which decreased non-significantly for prolonged sedentary behavior. For each 2 h increment per day in sedentary behavior, the mesor decreased on average −0.59 SD (95% CI: −0.67, −0.50), amplitude: −0.75 (−0.84, −0.67), CQ: −0.74 (−0.83, −0.66), I < O: −0.85 (−0.98, −0.72) and R24: −0.15 (−0.25, -0.05) (Table 3). For prolonged sedentary behavior, similar associations were seen, with the mesor decreasing −0.45 SD per 2 hours/day additional prolonged sedentary behavior (−0.51, −0.39), amplitude: −0.44 (−0.50, −0.37), CQ: −0.42 (−0.49, −0.36), I < O: −0.67 (−0.76, −0.58) and R24: −0.03 (−0.10, 0.05). For both sedentary behavior and prolonged sedentary behavior, most associations were driven by both intra-individual as well as inter-individual changes over time. Inter-individual differences were slightly stronger for all RAR parameters except for the dichotomy index where intra-individual associations were slightly stronger. Survivors with more sedentary time (per additional 2 hours) were less likely to be in the second (OR: 0.48; 95% CI: 0.36, 0.65) or third tertile (OR: 0.47; 0.32, 0.70) of acrophase compared to the first tertile, indicating a lower likelihood of being active later at the day (Table 4). No differences were found between the third versus the second tertile (OR: 1.06; 0.74, 1.50). The direction and effect sizes of the ORs for prolonged sedentary behavior were comparable to those for sedentary time.

**Table 3.** Longitudinal associations of sedentary behavior and physical activity with diurnal rest-activity rhythms between 6 weeks and 60 months post-treatment in the study population of CRC survivors.

		Mesor^a^ Mean activity level		Amplitude^a^ Difference between activity peak and mesor		Circadian Quotient^a^ Peak in activity adjusted for mean activity levels		Dichotomy index^a^ Difference between day and nighttime activity		24-h autocorrelation^a^ Consistency of rhythm across days	
		β^b^	95% CI	β^b^	95% CI	β^b^	95% CI	β^b^	95% CI	β^b^	95% CI
Total sedentary behavior (2 h/day) *(MOX)*	Adjusted^c,d^	**−0.59**	**(−0.67, −0.50)**	**−0.75**	**(−0.84, −0.67)**	**−0.74**	**(−0.83, −0.66)**	**−0.85**	**(−0.98, −0.72)**	**−0.15**	**(−0.25, −0.05)**
	Intra^e^	**−0.49**	**(−0.58, −0.40)**	**−0.62**	**(−0.70, −0.53)**	**−0.61**	**(−0.70, −0.51)**	**−1.31**	**(−1.45, −1.17)**	0.01	(−0.13, 0.15)
	Inter^f^	**−0.64**	**(−0.75, −0.53)**	**−0.78**	**(−0.89, −0.68)**	**−0.77**	**(−0.88, −0.67)**	**−0.81**	**(−0.94, −0.69)**	**−0.31**	**(−0.45, −0.17)**
Prolonged sedentary behavior (2 h/day) *(MOX)*	Adjusted^c,d^	**−0.45**	**(−0.51, −0.39)**	**−0.44**	**(−0.50, −0.37)**	**−0.42**	**(−0.49, −0.36)**	**−0.67**	**(−0.76, −0.58)**	−0.03	(−0.10, 0.05)
	Intra^e^	**−0.34**	**(−0.41, −0.27)**	**−0.35**	**(−0.42, −0.28)**	**−0.34**	**(−0.41, −0.26)**	**−1.10**	**(−1.20, −1.00)**	0.05	(−0.05, 0.15)
	Inter^f^	**−0.54**	**(−0.62, −0.47)**	**−0.47**	**(−0.55, −0.38)**	**−0.44**	**(−0.53, −0.36)**	**−0.72**	**(−0.80, −0.64)**	−0.10	(−0.21, 0.00)
Standing behavior (h/day) *(MOX)*	Adjusted^c,d^	**0.41**	**(0.36, 0.47)**	**0.40**	**(0.34, 0.46)**	**0.39**	**(0.32, 0.45)**	**0.59**	**(0.50, 0.69)**	−0.06	(−0.13, 0.01)
	Intra^e^	**0.39**	**(0.33, 0.45)**	**0.38**	**(0.31, 0.44)**	**0.36**	**(0.29, 0.43)**	**0.88**	**(0.78, 0.98)**	**−0.14**	**(−0.23, −0.05)**
	Inter^f^	**0.40**	**(0.32, 0.48)**	**0.37**	**(0.28, 0.45)**	**0.35**	**(0.27, 0.44)**	**0.49**	**(0.40, 0.58)**	0.05	(−0.05, 0.16)
Total physical activity (h/day) *(MOX)*	Adjusted^c,d^	**1.19**	**(1.11, 1.27)**	**1.24**	**(1.15, 1.32)**	**1.18**	**(1.09, 1.28)**	**1.11**	**(0.96, 1.27)**	**0.60**	**(0.46, 0.73)**
	Intra^e^	**1.07**	**(0.97, 1.16)**	**1.11**	**(1.01, 1.20)**	**1.05**	**(0.94, 1.16)**	**1.54**	**(1.35, 1.74)**	**0.46**	**(0.28, 0.63)**
	Inter^f^	**1.28**	**(1.19, 1.38)**	**1.24**	**(1.13, 1.34)**	**1.17**	**(1.06, 1.28)**	**0.98**	**(0.83, 1.14)**	**0.68**	**(0.52, 0.84)**
Light-intensity physical activity (h/day) (*SQUASH)*	Adjusted^c^	0.03	(−0.00, 0.06)	0.01	(−0.02, 0.04)	0.01	(−0.02, 0.04)	0.02	(−0.02, 0.07)	−0.02	(−0.06, 0.02)
	Intra^e^	0.03	(−0.00, 0.06)	0.02	(−0.02, 0.05)	0.02	(−0.02, 0.06)	0.05	(−0.01, 0.11)	−0.01	(−0.06, 0.04)
	Inter^f^	0.04	(−0.02, 0.10)	−0.01	(−0.06, 0.05)	−0.01	(−0.07, 0.05)	−0.00	(−0.06, 0.06)	−0.04	(−0.10, 0.03)
Moderate-to-vigorous-intensity-physical activity (h/day) *(SQUASH)*	Adjusted^c,d^	**0.18**	**(0.14, 0.22)**	**0.21**	**(0.18, 0.25)**	**0.21**	**(0.17, 0.25)**	**0.13**	**(0.07, 0.19)**	**0.10**	**(0.05, 0.15)**
	Intra^e^	**0.15**	**(0.11, 0.19)**	**0.17**	**(0.12, 0.21)**	**0.16**	**(0.11, 0.21)**	**0.10**	**(0.01, 0.18)**	0.06	(−0.01, 0.12)
	Inter^f^	**0.25**	**(0.18, 0.32)**	**0.31**	**(0.24, 0.37)**	**0.30**	**(0.24, 0.37)**	**0.16**	**(0.08, 0.23)**	**0.16**	**(0.09, 0.24)**

Values in bold are statistically significant (*P* < 0.05).

a. Each diurnal rest-activity rhythm parameter was included in the model per standard deviation (SD) increase. This SD was calculated by summing the SDs of each diurnal rest-activity rhythm parameter at each time- point, and then dividing this number by 5 (number of time points). The SDs were as follows: mesor 0.17, amplitude 0.18, circadian quotient 0.04, dichotomy index 0.11, and 24 h-autocorrelation coefficient 0.11.

b. The β-coefficients indicate the overall longitudinal difference in the outcome score using linear mixed models per 2 hours increase in sedentary behavior variables, and per 1 h increase in physical activity variables.

c. Linear mixed-models adjusted for sex (male/female), age at enrollment (years), time since end of treatment (days), neo-adjuvant chemotherapy and/or radiotherapy (yes/no), adjuvant therapy chemotherapy (yes/no), comorbidities (0, 1, ≥2), BMI (kg/m^2^), stoma (yes/no), smoking (former, current, never), employment status (yes/no), and alcohol intake (g/day).

d. A random slope was added to the model when the model improved statistically significantly using a likelihood-ratio test

e. The β-coefficients indicate the average change in the outcome score over time when total sedentary behavior and prolonged sedentary increases with 2 hours per day or when standing, total physical activity, LPA and VMPA increase with 1 hour per day between time points from 6 weeks to 60 months post-treatment within individuals.

f. The β-coefficients indicate the average difference in the outcome score between individuals differing when total sedentary behavior and prolonged sedentary increases with 2 hours per day or when standing, total physical activity, LPA and VMPA increase with 1 hour per day between time points from 6 weeks to 60 months post-treatment.

**Table 4.** Longitudinal associations of sedentary behavior and physical activity with acrophase between 6 weeks and 60 months post-treatment in the study population of CRC survivors.

		Acrophase *(Tertile 1 as reference)*		Acrophase *(Tertile 2 as reference)*	
		OR^a^	95% CI of OR	OR^a^	95% CI of OR
Sedentary behavior (2 h/day) *(MOX)*	Tertile 1	REF	REF	**2.94**	**(1.95, 4.42)**
	Tertile 2	**0.48**	**(0.36, 0.65)**	REF	REF
	Tertile 3	**0.47**	**(0.32, 0.70)**	1.06	(0.74, 1.50)
Prolonged sedentary behavior (2 h /day) *(MOX)*	Tertile 1	REF	REF	**2.46**	**(1.82, 3.32)**
	Tertile 2	**0.53**	**(0.43, 0.66)**	REF	REF
	Tertile 3	**0.49**	**(0.36, 0.66)**	1.00	(0.76, 1.32)
Standing behavior (h/day) *(MOX)*	Tertile 1	REF	REF	**0.68**	**(0.52, 0.87)**
	Tertile 2	**1.26**	**(1.04, 1.53)**	REF	REF
	Tertile 3	**1.34**	**(1.02, 1.76)**	1.01	(0.79, 1.28)
Total physical activity (h/day) *(MOX)*	Tertile 1	REF	REF	**0.49**	**(0.31, 0.76)**
	Tertile 2	**1.81**	**(1.27, 2.57)**	REF	REF
	Tertile 3	1.05	(0.65 1.71)	**0.59**	**(0.39, 0.91)**
Light-intensity physical activity (h/day) *(SQUASH)*	Tertile 1	REF	REF	1.02	(0.88, 1.17)
	Tertile 2	0.98	(0.87, 1.10)	REF	REF
	Tertile 3	1.00	(0.87, 1.17)	1.03	(0.90, 1.17)
Moderate-to-vigorous-intensity-physical activity (h/day) *(SQUASH)*	Tertile 1	REF	REF	1.09	(0.91, 1.29)
	Tertile 2	0.94	(0.82, 1.08)	REF	REF
	Tertile 3	0.99	(0.82, 1.19)	1.04	(0.88, 1.23)

Values in bold are statistically significant (*P* < 0.05).

a. The OR indicates the overall odds of being in a given tertile relative to the odds of being in the reference category for an increase of 2 hours in sedentary behavior variables, and per 1 hour increase in physical activity variables. Abbreviations: LPA, light-intensity physical activity; MVPA, moderate-to-vigorous-intensity-physical activity; REF, reference; OR, odds ratio; the ranges for the various tertiles are, tertile 1 (01:23:35 - 13:51:20), tertile 2 (13:51:28 - 14:42:40) and tertile 3 (14:42:55 - 20:10:02); the median values for the tertiles are: tertile 1 (13:17:50), tertile 2 (14:17:11), tertile 3 (15:13:50).

### Standing and Total Physical Activity

More time spent in objectively measured standing was significantly associated with higher values for all RAR parameters besides the R24. More time spent in total physical activity, was significantly associated with higher values for all RAR parameters. For each hour per day increase in standing, the mesor increased 0.41 SD (0.36, 0.47), amplitude: 0.40 (0.34, 0.46), CQ: 0.39 (0.32, 0.45), I < O: 0.59 (0.50, 0.69) and the R24 decreased non-significantly: −0.06 (−0.13, 0.01). Stronger associations were observed for total physical activity compared to standing, where a 1 hour increase in total physical activity per day was longitudinally associated with a higher mesor: 1.19 (1.11, 1.27), amplitude: 1.24 (1.15, 1.32), CQ: 1.18 (1.09, 1.28), I < O: 1.11 (0.96, 1.27), and R24: 0.60 (0.46, 0.73). For standing, the associations were driven by both intra-individual and inter-individual differences with comparable magnitude of associations, however, for R24 only an intra-individual significant association was observed and for I < O, the intra-individual association was stronger than the inter-individual association. For total physical activity, both inter and intra-driven associations were observed with generally stronger inter-individual associations except for the I < O where a stronger intra-individual association was observed. Survivors with more standing time (per additional 1 h) were more likely to be in the second (OR: 1.26; 1.04, 1.53) or third tertile (OR: 1.34; 1.02, 1.76) of the acrophase compared to the first tertile (Table 4). No significant differences were observed between tertile two and tertile three (OR: 1.01; 0.79, 1.28). Survivors with more physical activity (per additional 1 h) were more likely to have a mid-day acrophase (tertile two, OR: 1.81; 1.27, 2.57) while there was no difference between the third tertile (1.05; 0.65, 1.71) and first tertile. When the reference category was the second tertile, there was a lower odds of being in the third tertile (0.59; 0.39, 0.91).

### Light-Intensity-Physical Activity and Moderate-to-Vigorous-Physical Activity

We found no significant longitudinal associations between self-reported LPA (overall, inter or intra-individual) and RAR parameters and effect estimates were small. Higher MVPA was longitudinally associated with higher mesor: 0.18 SD (0.14, 0.22), amplitude: 0.21 (0.18, 0.25), CQ: 0.21 (0.17, 0.25), I < O: 0.13 (0.07, 0.19), and R24: 0.10 (0.05, 0.15). The overall associations were driven by both intra-individual and inter-individual associations, although more-pronounced inter-individual associations were found. LPA and MVPA were not significantly associated with the acrophase.

### Interaction and Sensitivity Analyses

For some of the described overall associations, significant interaction terms were observed with time since end of treatment. In case of significant interaction, effects of each post-treatment time point are visualized in Supplementary Figure 2. Generally, observed associations for CQ (MVPA), amplitude and dichotomy index seemed to be stronger at 6 weeks. For the mesor and CQ associations tended to be strongest at 2 to 5 years posttreatment. For the 24 h-autocorrelation in regard to total physical activity, associations were strongest at 2 years posttreatment. Regarding the acrophase, no consistent significant interaction effects with time since end of treatment were observed. No significant interactions were found for sex and BMI. For the sensitivity analyses with additional adjustment for napping, (prolonged) sedentary behavior and MVPA, we found similar associations as compared to the main results (Supplementary Figures 3–6). We performed a sensitivity analysis also adjusting for cancer stage and found no differences in associations. Finally, with regards to the time lag model, the associations of sedentary behavior and physical activity with RAR parameters were attenuated with many associations becoming nonsignificant (Supplementary Figure 7).

## Discussion

We examined longitudinal associations of sedentary behavior, standing, and physical activity parameters with objectively measured RAR parameters in a cohort of CRC survivors from 6 weeks up to 5 years post-treatment. Generally, we observed that higher prolonged sedentary behavior and lower physical activity were longitudinally associated with lower RAR parameters, with associations driven by both intra-individual changes over time and between individual differences. This was in line with our hypothesis predicting that higher (prolonged) sedentary behavior and lower standing and physical activity are associated with a less robust RAR. In contrast, LPA was not associated with changes in RAR parameters. Our findings regarding acrophase showed that less sedentary behavior and more standing and physical activity were associated with a higher likelihood of a mid-day peak in activity. No significant associations were observed between LPA and MVPA and the acrophase.

These results highlight that both physical activity and standing behaviors have the potential to cause more pronounced RAR parameters, which may potentially improve outcomes such as fatigue and quality of life. Therefore, remaining active during and post CRC treatment remains crucial in ensuring that activity rhythms are maintained (Berger et al., 2010). Activity recommendations for cancer survivors include reducing prolonged sedentary behavior and encouraging more physical activity (WHO, 2020, Jochem and Leitzmann, 2022, Smit et al., 2024). However, despite our observed associations, these associations were attenuated in the time lag model, suggesting that associations are potentially reciprocal. The directionality of the relations therefore requires further investigation. In addition, the lack of overall associations found for LPA with RAR parameters also needs to be addressed. Potentially LPA does not associate with RAR parameters since LPA is in between sedentary and total physical activity, not driving RAR rhythms up or down. In addition, due to the self-reported nature of the LPA, more error was to be expected due to over or underestimation.

A previous study in patients with lung cancer by Chen et al. found more favorable R24 and I < O when patients performed activity particularly LPA for at least 295 min/day (Chen et al., 2016). This was partially in line with our findings, showing that an increased total physical activity was associated with more favorable R24 and I < O, indicative of a more robust RAR. We, however, did not find any significant associations for LPA with R24 and I < O. Another study in breast cancer survivors revealed marked differences in mesor and amplitude compared to a healthy control group with similar job categories (i.e. teaching, office, or housework), which they largely attributed to their differences in daily activity levels (Roveda et al., 2019). Associations between sedentary behavior and physical activity with acrophase have, to the best of our knowledge, not been investigated in any cancer population and our findings need to be replicated.

Previous studies have however, showed the link between improved rhythms and increased patient outcomes such as decreased fatigued and improved QoL (Berger et al., 2010, Chong et al., 2024). We observed that there are beneficial effects on RAR rhythms by lowered sedentary behavior and higher standing levels. Preceding research, including work of our research group, has shown that CRC survivors may benefit from low levels of prolonged sedentary behavior and, unlike our findings, also engage more in forms of LPA ultimately leading to more positive health-related outcomes (Blair et al., 2014, Swain et al., 2020, van Roekel et al., 2020, Kenkhuis et al., 2021, Jochem and Leitzmann, 2022). A possible explanation for this could be that an older individual, who may suffer from more comorbidities, may not be able to engage in strenuous levels of activity. Therefore, decreasing sedentary behavior and improving LPA and standing behavior, being more accessible, may have positive effects on health-related outcomes including physical functioning and fatigue. In addition, our group has shown that substituting sedentary time with for example standing or physical activity was significantly associated with better physical functioning in CRC survivors (van Roekel et al., 2016a).

Our finding that stage I-III CRC survivors with more sedentary behavior and less standing or physical activity had more disrupted RAR parameters can have important clinical implications. Disrupted RAR has been associated with worse fatigue, shorter survival, and reduced health-related quality of life in patients with metastatic CRC patients (Mormont et al., 2000, Innominato et al., 2009). In addition, disrupted RAR has been associated with inflammation and immune dysregulation in various animal models. Hence, interventions to enhance circadian regulation can have important clinical significance (Logan and Sarkar, 2012, Lawther et al., 2022). Our findings highlight the potential for interventions targeting prolonged sedentary behavior and improving standing and physical activity behavior for patients with CRC. Next to reducing sitting time and increasing exercise behavior, light therapy and timing of food intake have shown promising results to improve circadian rhythms in patients with metastatic disease (75).

An important strength of this study is its prospective design, featuring repeated and extensive measurements over time within a large cohort of CRC survivors including data on relevant covariates. In addition, the 5-year follow-up period enables assessment of long-term changes. The measurements that were used to collect the data for this study were done both with validated instruments, including the MOX accelerometer and SQUASH. The use of linear mixed models enabled us to distinguish between inter-and intra-individual associations, and to handle missing data under the assumption that missing data are completely at random. The high response rates over time and the minimal dropouts due to death suggest little evidence for missing data not at random. Instead of linear mixed methods, the Hidden Markov Model (HMM) could be another approach for modeling rest-activity time series by distinguishing between behavioral states (Witowski et al., 2014). However, the HMM makes the assumption that the next state depends only on the current state, making it less able to identify larger relationships in the data, and applying the model to larger datasets can become computationally challenging (Dimri et al., 2024).

There were also some limitations to be noted. Due to the observational nature of this study, no causal conclusions can be made. Additional studies, such as randomized controlled trials are necessary to investigate whether increasing physical activity and decreasing sedentary behavior will improve RAR rhythms. The SQUASH, though being a validated questionnaire, is a self-reported measure which is prone to recall bias (Wagenmakers et al., 2008, Nicolaou et al., 2016, Terpstra et al., 2024). There is likely also a certain degree of selection bias at recruitment and during follow-up, since individuals who are healthier and are better educated are more likely to continue to participate in the study (Kripalani et al., 2021). Another limitation is the inherent correlation between physical activity variables and RAR parameters, particularly in variables such as the mesor, reflecting mean activity levels (Supplementary Figure 1). Despite this, our study examines how independent behavioral components, such as physical activity and sedentary time, are associated with changes in RAR parameters over time. While variables such as the mesor correlate highly with total physical activity, other RAR parameters, such as the dichotomy index and acrophase, offer unique insights into rhythmicity beyond overall activity levels or sedentary behavior. By distinguishing these relationships, our study enhances understanding of how physical activity and sedentary time independently relate to RARs. Further research should explore how these rhythms influence clinical outcomes. Finally, we cannot rule out the possibility of some chance findings due to the high number of statistical tests performed.

## Conclusion

Our findings show that in the first 5 years after CRC treatment, lower sedentary behavior and higher standing time and physical activity are associated with higher RAR parameters indicating a more pronounced rhythm. Next steps should focus on the development and evaluation of interventions aimed at improving RAR through reducing sedentary behavior and increasing physical activity and testing if such interventions can improve health-related outcomes in the period after CRC treatment.

## Supplemental Material

sj-docx-1-jbr-10.1177_07487304251363674 – Supplemental material for Longitudinal Associations of Sedentary Behavior and Physical Activity With Diurnal Rest-Activity Rhythms in Survivors of Colorectal Cancer Up to 5 Years Post-Treatment Supplemental material, sj-docx-1-jbr-10.1177_07487304251363674 for Longitudinal Associations of Sedentary Behavior and Physical Activity With Diurnal Rest-Activity Rhythms in Survivors of Colorectal Cancer Up to 5 Years Post-Treatment by Koen G. Frenken, Marvin Y. Chong, Stéphanie O. Breukink, Maryska Janssen-Heijnen, Eric T.P. Keulen, Joop Konsten, Wouter Bijnens, Laurien M. Buffart, Kenneth Meijer, Frank A.J.L. Scheer, Karen Steindorf, Judith de Vos-Geelen, Matty P. Weijenberg, Martijn J.L. Bours and Eline H. van Roekel in Journal of Biological Rhythms
