# Supplementary material for: Longitudinal Associations of Sedentary Behavior and Physical Activity With Diurnal Rest-Activity Rhythms in Survivors of Colorectal Cancer Up to 5 Years Post-Treatment
Source: J Biol Rhythms. 2025 Oct 1;40(6):528–46. doi: 10.1177/07487304251363674 (PMC12572352; doi:10.1177/07487304251363674)
Supplement: sj-docx-1-jbr-10.1177_07487304251363674 – Supplemental material for Longitudinal Associations of Sedentary Behavior and Physical Activity With Diurnal Rest-Activity Rhythms in Survivors of Colorectal Cancer Up to 5 Years Post-Treatment [file sj-docx-1-jbr-10.1177_07487304251363674.docx]

Supplementary Figures

|  | **SED** | **PROSED** | **STAND** | **TPA** | **LPA** | **MVPA** | **MESOR** | **AMP** | **CQ** | **I<O** | **R24** | **ACR** |
| --- | --- | --- | --- | --- | --- | --- | --- | --- | --- | --- | --- | --- |
| **SED** | 1.00 |  |  |  |  |  |  |  |  |  |  |  |
| **PROSED** | 0.65 | 1.00 |  |  |  |  |  |  |  |  |  |  |
| **STAND** | -0.73 | -0.51 | 1.00 |  |  |  |  |  |  |  |  |  |
| **TPA** | -0.63 | -0.68 | 0.46 | 1.00 |  |  |  |  |  |  |  |  |
| **LPA** | -0.05 | -0.06 | 0.17 | 0.04 | 1.00 |  |  |  |  |  |  |  |
| **MVPA** | -0.21 | -0.17 | 0.13 | 0.45 | 0.02 | 1.00 |  |  |  |  |  |  |
| **MESOR** | -0.43 | -0.54 | 0.43 | 0.75 | 0.16 | 0.40 | 1.00 |  |  |  |  |  |
| **AMP** | -0.54 | -0.44 | 0.37 | 0.74 | 0.12 | 0.44 | 0.75 | 1.00 |  |  |  |  |
| **CQ** | -0.53 | -0.41 | 0.35 | 0.70 | 0.12 | 0.42 | 0.69 | 0.99 | 1.00 |  |  |  |
| **I<O** | -0.65 | -0.76 | 0.58 | 0.63 | 0.04 | 0.16 | 0.27 | 0.28 | 0.26 | 1.00 |  |  |
| **R24** | -0.22 | -0.18 | 0.03 | 0.48 | -0.12 | 0.27 | 0.40 | 0.53 | 0.53 | 0.14 | 1.00 |  |
| **ACR** | -0.47 | -0.58 | 0.37 | 0.38 | -0.04 | 0.01 | 0.01 | -0.03 | -0.04 | 0.79 | -0.04 | 1.00 |

**Supplementary Figure 1**. Pearson correlation coefficients at 6 weeks post-treatment between sedentary behavior and physical activity parameters and diurnal rest-activity rhythm parameters. Abbreviations: SED, sedentary behavior; PROSED, prolonged sedentary behavior; STAND, standing behavior; TPA, total physical activity; LPA, light intensity physical activity; MVPA, moderate- to-vigorous physical activity; AMP, amplitude; CQ, circadian quotient; R24, 24h-autocorrelation; I<O, dichotomy index ACR, acrophase.

**Supplementary Figure 2**. Figure illustrating significant interaction of overall longitudinal associations between sedentary behavior and physical activity parameters and diurnal rest-activity rhythm parameters with time since end of treatment analyzed as dummy variables for each timepoint. Effects are shown separately for each post-treatment time point, and significantly different associations as compared to 6 weeks post-treatment are indicated with an asterisk. Significant interaction was observed between sedentary behavior and physical activity parameters with time since treatment analyzed continuously (per 6 months) in relation to RAR parameters. Abbreviations: PT, post-treatment; SED, sedentary behavior; PROSED, prolonged sedentary behavior; STAND, standing behavior; TPA, total physical activity; LPA, light intensity physical activity; MVPA, moderate- to-vigorous physical activity; AMP, amplitude; CQ, circadian quotient; R24, 24-h autocorrelation; I<O, dichotomy index.

**Supplementary Figure 3.** Figure comparing the main results of the overall associations between sedentary behavior and physical activity variables and diurnal rest-activity rhythm parameters, with and without additional adjustment for presence of napping during the day (yes/no). Abbreviations: SED, sedentary behavior; PROSED, prolonged sedentary behavior; STAND, standing behavior; TPA, total physical activity; LPA, light-physical activity; MVPA, moderate-to-vigorous physical activity.

**Supplementary Figure 4.** Figure comparing the main results of the overall associations between sedentary behavior, standing and LPA variables and diurnal rest-activity rhythm parameters, with and without additional adjustment for MVPA. Abbreviations: SED, sedentary behavior; PROSED, prolonged sedentary behavior; STAND, standing behavior; TPA, total physical activity; LPA, light-physical activity.

**Supplementary Figure 5.** Figure comparing the main results of the overall associations between physical activity variables and diurnal rest-activity rhythm parameters, with and without additional adjustment for sedentary behavior. Abbreviations: TPA, total physical activity; MVPA, moderate-to-vigorous physical activity.

**Supplementary Figure 6.** Figure comparing the main results of the overall associations between sedentary behavior and physical activity variables and diurnal rest-activity rhythm parameters, and similar associations from a time-lag model (exposure at one post-treatment time point combined with outcomes at the next post-treatment time point). Abbreviations: SED, sedentary behavior; PROSED, prolonged sedentary behavior; STAND, standing behavior; TPA, total physical activity; LPA, light-physical activity; MVPA, moderate-to-vigorous physical activity.

**Supplementary Figure 7.** Forest plot comparing the increase of sedentary behavior and physical activity parameters with the main associations for the acrophase compared to the associations from the sensitivity analysis: napping, MVPA, sedentary behavior and the time lag model. An odds ratio of 1 equals that there is no higher odds of ending up in a different tertile as compared to the reference tertile, while a OR greater than 1 indicates a higher odds of ending up in that tertile and a OR lower than 1 indicates a lower odds of ending up in another tertile, compared to the reference tertile. Abbreviations: adj, adjustment; LPA, light-physical activity; MVPA, moderate-to-vigorous physical activity; REF, reference; SED, sedentary.

**Supplementary Table 8.** Clinical characteristics and treatment characteristics in the study population of CRC survivors.

| **Characteristics** | **6 weeks post- treatment**  **(*n* = 268)^1^** | **6 months post- treatment**  **(*n* = 254)^1, 2^** | **12 months post- treatment**  **(*n* = 214)^1, 2^** | **24 months post- treatment**  **(*n* =138)^1, 2^** | **60 months post- treatment**  **(*n* =77)^1, 2^** |
| --- | --- | --- | --- | --- | --- |
| **Tumor site, *n* (%)** |  |  |  |  |  |
| Colon | 86 (32.1) | 73 (28.7) | 69 (32.2) | 44 (31.9) | 23 (29.9) |
| Sigmoid | 81 (30.2) | 87 (34.3) | 65 (30.4) | 38 (27.5) | 24 (31.2) |
| Rectum | 101 (37.7) | 94 (37.0) | 80 (37.4) | 56 (40.6) | 30 (39.0) |
| **Treatment for primary colon tumor, *n* (%)** |  |  |  |  |  |
| No Treatment | 2 (2.3) | 0 | 1 (1.4) | 1 (2.3) | 0 |
| Surgery alone | 62 (72.1) | 59 (80.8) | 51 (73.9) | 33 (75.0) | 18 (78.3) |
| Surgery and chemotherapy | 22 (25.6) | 14 (19.2) | 17 (24.6) | 10 (22.7) | 5 (21.7) |
| **Treatment for primary sigmoid tumor, *n* (%)** |  |  |  |  |  |
| No Treatment | 7 (8.6) | 7 (8.1) | 5 (7.7) | 1 (2.6) | 2 (8.3) |
| Surgery alone | 47 (58.0) | 48 (55.2) | 38 (58.5) | 24 (63.2) | 18 (75.0) |
| Radiotherapy alone | 1 (1.2) | 1 (1.2) | 0 | 0 | 0 |
| Surgery and radiotherapy | 0 | 1 (1.2) | 0 | 0 | 0 |
| Surgery and chemotherapy | 26 (32.1) | 30 (34.5) | 22 (33.9) | 13 (34.2) | 4 (16.7) |
| **Treatment for primary rectal tumor, *n* (%)** |  |  |  |  |  |
| No Treatment | 3 (3.0) | 4 (4.3) | 5 (6.3) | 5 (8.9) | 1 (3.3) |
| Surgery alone | 27 (26.7) | 23 (24.5) | 17 (21.3) | 11 (19.6) | 8 (26.7) |
| Surgery and chemotherapy | 2 (2.0) | 2 (2.1) | 1 (1.3) | 1 (1.8) | 1 (3.3) |
| Surgery and radiotherapy | 18 (17.8) | 18 (19.2) | 15 (18.8) | 9 (16.1) | 5 (16.7) |
| Radiotherapy and chemotherapy | 18 (17.8) | 14 (14.9) | 14 (17.5) | 8 (14.3) | 5 (16.7) |
| Surgery, radiotherapy and chemotherapy | 33 (32.7) | 33 (35.1) | 28 (35.0) | 22 (39.3) | 10 (33.3) |
| **Chemotherapy, *n* (%)** |  |  |  |  |  |
| Neoadjuvant | 49 (18.3) | 45 (17.7) | 41 (19.2) | 29 (21.0) | 14 (18.2) |
| Adjuvant | 74 (27.6) | 65 (25.6) | 62 (29.0) | 37 (26.8) | 20 (26.0) |
| **Cancer stage, *n* (%)** |  |  |  |  |  |
| Stage I | 85 (31.7) | 83 (32.7) | 72 (33.6) | 44 (31.9) | 24 (31.2) |
| Stage II | 68 (25.4) | 61 (24.0) | 50 (23.4) | 36 (26.1) | 25 (32.5) |
| Stage III | 115 (42.9) | 110 (43.3) | 92 (43.0) | 58 (42.0) | 28 (36.4) |

^1^Percentages may not add up to 100% due to rounding.

^2^Response rates for the follow-up time points up to 24-months were all above 90% and for 60 months 63%. The decreasing absolute numbers are largely due to the fact that participants had not yet reached all post-treatment follow-up time points at the time of data acquisition in July 2018 for measurements at 6 weeks to 24 months post-treatment and in October 2021 for 60 months post-treatment.

**Supplementary Table 9** – Comparison of exposure and outcome characteristics between individuals at 24 months post-treatment who were still in the study at 60 months post-treatment versus individuals who dropped out between 24 months and 60 months post

| **Characteristics** | **Individuals at 24 months post-treatment who were still in the study at 60 months post-treatment (N = 58)** | **Individuals at 24 months post-treatment who dropped out after this time point**  **(n = 80)** |
| --- | --- | --- |
| **Sedentary behavior and physical activity variables (mean ± SD) / (median – 25^th^ percentage – 75^th^ percentage)**  **(hours / day)** | | |
| Sedentary behavior | 10.2 ± 1.3 | 10.3 ± 1.5 |
| Prolonged sedentary behavior | 4.6 ± 1.9 | 4.4 ± 1.8 |
| Standing behavior | 3.3 ± 1.0 | 3.5 ± 1.1 |
| Total physical activity | 1.8 ± 0.7 | 1.7 ± 0.6 |
| LPA | 1.5 (0.5-3.0) | 1.4 (0.5-3.1) |
| MVPA | 1.5 (0.9 – 2.7) | 1.4 (0.6-2.6) |
| **Diurnal rest activity parameters (mean ± SD) / (median – 25^th^ percentage – 75^th^ percentage)** | | |
| Mesor | 3.8 ± 0.2 | 3.8 ± 0.2 |
| Amplitude | 0.6 ± 0.2 | 0.6 ± 0.2 |
| Acrophase (clock time (hh:mm) | 14:18 (13:48-15:08) | 13:56 (13:23 – 14:41) |
| Circadian quotient | 0.2 ± 0.0 | 0.2 ± 0.0 |
| Dichotomy index | 0.9 ± 0.1 | 0.9 ± 0.1 |
| 24h - autocorrelation**^2^** | 0.2 ± 0.1 | 0.2 ± 0.1 |
